# Correlation Between Serum β2-GPI/oxLDL and the Risk of Cerebral Infarction in Patients with T2DM

**DOI:** 10.3389/fsurg.2022.930701

**Published:** 2022-07-11

**Authors:** Wending Kuang, Yi Li, Gang Liu, Yang Zhang, Gang Chen, Bang Luo, Shuangyu Kuang

**Affiliations:** ^1^ The Second Affiliated Hospital, Department of Neurology, Hengyang Medical School, University of South China, Hengyang, China; ^2^ Department of Diagnosis, Hengyang Medical School, University of South China, Hengyang, China; ^3^ The Second Affiliated Hospital, Department of Endocrinology, Hengyang Medical School, University of South China, Hengyang, China; ^4^ The Second Affiliated Hospital, Department of GCP, Hengyang Medical School, University of South China, Hengyang, China

**Keywords:** oxLDL, β2-GPI, cerebral infarction, type 2 diabetes, T2DM

## Abstract

**Objective:**

This study aims to study the correlation between serum β2-glycoprotein I (β2-GPI)/oxidized low-density lipoprotein (oxLDL) and the risk of cerebral infarction in patients with type 2 diabetes (T2DM).

**Methods:**

From January 2019 to March 2021, 56 patients with T2DM combined with cerebral infarction were chosen as a diabetic cerebral infarction (DCI) group, and 60 patients with simple T2DM were chosen as a T2DM group. In addition, 60 healthy volunteers were recruited as a control group. The essential information of each group was collected, and the serum β2-GPI/oxLDL and inflammatory factor levels in each group were compared. The clinical factors that affect the risk of ischemic cerebral infarction in patients with T2DM were analyzed by a logistic model.

**Results:**

Compared with the control group, the level of serum β2-GPI/oxLDL in the T2DM and DCI groups increased significantly, *P *< 0.001. Compared with the T2DM group, the serum β2-GPI/oxLDL level in the DCI group increased significantly, *P *< 0.05. The result of *Pearson’s* correlation analysis showed that serum β2-GPI/oxLDL was positively correlated with total cholesterol, triglycerides, fasting blood glucose, 2-h postprandial blood glucose, glycosylated hemoglobin, interleukin-6, and tumor necrosis factor (TNF)-α (all *P*’s* *< 0.05). Serum TNF-α and β2-GPI/oxLDL were independent risk variates for DCI (*P *< 0.05). Based on the receiver operating characteristic curve analysis, the values of the area under the curve for TNF-α, serum β2-GPI/oxLDL, and the combined diagnosis of DCI were 0.653 (0.552–0.753), 0.680 (0.583–0.777), 0.739 (0.647–0.831), respectively.

**Conclusion:**

In DCI patients, the levels of serum oxLDL/β2-GPI are significantly increased. Serum oxLDL/β2-GPI is an independent risk factor that affects the occurrence of DCI. In addition, the serum β2-GPI/oxLDL level implicates the lipid metabolism and inflammatory status of the internal environment of DCI patients to a certain extent.

## Introduction

Type 2 diabetes mellitus (T2DM) is a chronic disease characterized by progressive insulin resistance and hyperglycemia and is proposed as an independent risk factor for cerebrovascular disease (1–2). T2DM is the most common type of diabetes and is also known as adult-onset diabetes because it occurs mostly in adults. The disease is caused by various causes that lead to insufficient insulin secretion in the body or the body cannot use insulin effectively, resulting in a continuous increase in blood sugar levels, eyes, and other organs. Mild forms of diabetes can be controlled with dietary intervention without medication (3). The largest autopsy study to date of cerebral infarction deceased confirmed a 1.57% increased risk of cerebral infarction associated with T2DM.

At present, cerebral infarction in T2DM is diagnosed mainly by imaging, but the imaging examination has certain limitations. It is of great significance to patients with T2DM if there are suitable serum indicators that accurately predict the risk of cerebral infarction. Low-density lipoprotein is a lipoprotein particle that carries cholesterol into peripheral tissue cells. When low-density lipoprotein, especially oxidized low-density lipoprotein (oxLDL), is in excess, the cholesterol it carries will accumulate on the arterial wall, which is easy to cause arteriosclerosis in the long run. Therefore, LDL is called “bad cholesterol.” oxLDL aggravates the inflammatory response and promotes the accumulation of cholesterol in lysosomes as well, eventually leading to cell death (4), which is a key factor in the occurrence of cardiovascular diseases. β2-Glycoprotein I (β2-GPI) activates platelets by interacting with cell surface phospholipids (phosphatidylserine, phosphatidylethanolamine) or platelet membrane receptors and exacerbates the progression of cardiovascular disease (5). oxLDL binds β2-GPI to form an oxLDL/β2-GPI complex, which induces atherosclerosis and the formation of foam cells. Studies have shown that elevated serum β2-GPI/oxLDL is associated with the occurrence of cerebral complications in T2DM patients (6). The purpose of this study was to deeply analyze the role of serum β2-GPI/oxLDL in T2DM complicated with cerebral infarction and to study the effect of serum β2-GPI/oxLDL in predicting cerebral infarction in T2DM patients.

## Clinical Data and Methods

### Clinical Data

From January 2019 to March 2021, a total of 56 patients with T2DM and cerebral infarction admitted to our hospital were included in the diabetic cerebral infarction (DCI) group, which includes 14 patients with cardio-embolism, 25 cases of large artery atherosclerotic, 11 cases of small artery occlusion type, and 6 cases of unknown etiology. There were 27 cases of anterior circulation infarction and 29 cases of posterior circulation infarction. A total of 60 patients only with T2DM were included in the T2DM group, and healthy volunteers without a history of diabetes and cerebral infarction who were age- and sex-matched with the above two groups of patients were included in the control group (*n* = 60). The average age of the DCI group was 65.86 ± 8.74 (43–81 years), with 33 males and 23 females. The average age of the T2DM group was 65.17 ± 10.55 (41–81 years), with 36 males and 24 females. The average age of the control group was 65.18 ± 9.50 (44–82 years), with 34 males and 26 females. Inclusion criteria are as follows: (1) the diagnostic criteria for T2DM conform to the “China Guidelines for the Prevention and Treatment of Type 2 Diabetes (2017 Edition)” (7); (2) the diagnostic criteria for cerebral infarction conform to the “Chinese Guidelines for Diagnosis and Treatment of Cerebral Infarction with Integrated Traditional Chinese and Western Medicine (2017)” (8), and cerebral infarction has occurred within 1 month; (3) 18–85 years old; (4) patients in the T2DM group had no history of cerebral infarction and clinical symptoms and signs of diabetic cerebral infarction; and (5) the physical, electrocardiogram, ultrasound, and biochemical examinations of the volunteers in the control group were all normal. Exclusion criteria are as follows: patients with cerebral hemorrhage on head imaging, gestational diabetes mellitus, diabetic macrovascular (including cardiovascular accident and lower extremity arterial disease) and microvascular (including diabetic nephropathy, diabetic retinopathy, and diabetic neuropathy) complications, type 1 diabetes, infectious disease, chronic liver disease, chronic kidney disease, malignant tumor, or any hereditary disease that affects lipid metabolism. This study was approved by the Ethics Review Board of our institution, and all participants provided written informed consent prior to the study. All methods were performed according to approved guidelines and regulations.

## Methods

### Clinical Data Collection

General information about the patient was collected, including age, gender, body mass index (BMI), duration of T2DM, history of cardiovascular disease (history of hypertension, family history of coronary heart disease, history of arrhythmia, family history of hyperlipidemia or diabetes mellitus history), smoking history, drinking history, and biochemical indicators (fasting blood glucose [PBG], 2-h postprandial blood glucose [2-h PG], fasting insulin [Fins], total cholesterol, triglycerides, high-density lipoprotein cholesterol [HDL-C], low-density lipoprotein cholesterol [LDL-C], and glycosylated hemoglobin [hemoglobin A1C, HbA1c]).

### Serum β2-GPI/oxLDL

The concentration of serum β2-GPI/oxLDL was determined by a sandwich enzyme-linked immunosorbent assay. First, blood samples were collected after 12 h of fasting, centrifuged at 1,500 r/min (radius: 8 cm) for 15 min, and stored at –80 °C. The polyclonal antibody against human β2-GPI was immobilized on a 96-well plate, and the polyclonal antibody against apolipoprotein B was used as the detection antibody. About 500 μl of serum was incubated at room temperature for 2 h, polyethylene glycol was added, and the samples were incubated overnight (4°C). The sample was centrifuged (10,000 r/min, radius: 8 cm, 20 min), and the obtained precipitate was resuspended in 0.01 mol/L PBS. β2-GPI antibody (2.5 μg/ml) was added to the samples, followed by incubation at 37°C for 2 h and then at 4°C overnight. The diluted samples (1:40) were added to the wells and blocked with 1% gelatin. BSA was added and incubated for 2 h. Standard β2-GPI/ox-LDL complexes were added to the wells and incubated overnight at 4°C to obtain a standard curve. Horseradish peroxidase-labeled goat antirabbit LDL polyclonal antibody was added to the wells, and TMBUS was added after incubation for 3 h (room temperature). The absorbance was read at 450 nm by using a microplate reader, and the serum oxLDL/β2-GPI complex concentration was calculated based on the standard curve.

### Serum Inflammatory Factors

An enzyme-linked immunosorbent assay kit (Diaclone, France) was used to detect tumor necrosis factor (TNF)-α and interleukin (IL)-6 in serum, the samples were detected by a Lablifeer/ew 2007, Varioskan LUX multimode microplate reader (CA, USA), and the concentrations of IL-6 and TNF-α in the sample were calculated based on the fitted concentration–absorbance curve. C-reactive protein (CRP) was detected by immuno-turbidimetry using a Hitachi 7020 automatic analyzer (Hitachi Kokusai Electric Inc., Tokyo, Japan).

### Statistical Methods

SPSS 20.0 software was used for data analysis; the data were tested for normality first, and the continuous variables conforming to the normal distribution were expressed as $\bar{x}\pm s$). Comparisons between groups were performed using an independent sample *t-*test or one-way ANOVA, and data with skewed normal distribution were expressed as M50 (P25, P75). Comparisons between groups were performed using the Mann–Whitney *U* or the Kruskal–Wallis test. The enumeration data were expressed in the form of cases (percentages), the comparison between groups was performed by the chi-square test, and Pearson’s analysis was used to measure the correlation of each index. Logistic model analysis of clinical factors affecting the risk of ischemic cerebral infarction in patients with T2DM was carried out, and the receiver operating characteristic (ROC) curve was used to analyze the diagnostic efficacy of serum β2-GPI/oxLDL for DCI. When *P* < 0.05, the data were statistically different.

## Results

### Clinical Data Comparison of Three Groups

There were no significant differences in age, gender, smoking history, drinking history, T2DM course, cardiovascular disease history, total cholesterol, triglyceride, and HDL-C among the three groups (*P *> 0.05). Compared with the control group, the BMI, LDL-C, PBG, 2-h PG, HbAlc, Fins, CRP, TNF-α, and IL-6 of the T2DM group and DCI group were significantly increased, with *P* values less than 0.05. Compared with those in the T2DM group, the TNF-α and CRP levels in the DCI group were significantly increased, and the *P* values were all less than 0.05, as shown in Table 1.

**Table 1:** Comparison of clinical data in three groups $\bar{x}\pm s$.

Indexes	T2DM group (*n* = 60)	DCI group (*n* = 56)	Control group (*n* = 60)	*F/χ2/Z*	*P* value
Age (year, M_50_[P_25_,P75])	65.17 ± 10.55	65.86 ± 8.74	65.18 ± 9.50	0.096	0.908
Male (cases, %)	36 (60.0)	33 (58.93)	34 (56.67)	0.143	0.931
Smoking history (cases, %)	20 (33.33)	18 (32.14)	21 (35.0)	1.320	0.517
History of alcohol intake (cases, %)	12 (20.0)	11 (19.64)	10 (16.67)	0.262	0.877
BMI (kg/m^2^, $\bar{x}\pm s$)	24.05 ± 1.63^a^	24.35 ± 1.63^a^	20.95 ± 1.74	70.460	<0.001
Course of T2DM (cases, %)					
≥10 years	44 (73.33)	34 (60.71)	–	2.094	0.148
<10 years	16 (26.67)	22 (39.29)	–		
History of cardiovascular disease (cases, %)					
coronary heart disease	25 (41.67)	23 (41.07)	–	0.004	0.948
Arrhythmia	31 (51.67)	34 (60.71)	–	0.963	0.327
Hypertension	50 (83.33)	47 (83.93)	–	0.007	0.931
hyperlipidemia	13 (21.67)	11 (19.64)	–	0.072	0.788
Blood index					
Total cholesterol (mmol/L, $\bar{x}\pm s$)	4.54 ± 1.14	4.49 ± 1.18	4.70 ± 1.30	0.494	0.611
Triglycerides (mmol/L, $\bar{x}\pm s$)	1.47 ± 0.41	1.54 ± 0.39	1.40 ± 0.43	1.593	0.206
LDL-C (mmol/L, M_50_[P_25_,P75])	2.56 (2.01,3.41)^a^	2.71 (1.83,3.35)^a^	2.16 (1.74,2.85)	8.157	0.017
HDL-C (mmol/L, $\bar{x}\pm s$)	1.01 (0.86,1.17)	0.94 (0.82,1.06)^a^	1.02 (0.86,1.22)	4.756	0.093
PBG (mmol/L, M_50_[P_25_,P75])	8.17 (7.20,9.36)^a^	8.30 (7.25,9.56)^a^	4.83 (4.19,5.60)	112.619	<0.001
2 h PG (mmol/L, M_50_[P_25_,P75])	15.50 (12.87,18.90)^a^	15.72 (12.59,18.67)^a^	6.23 (5.66,6.81)	117.718	<0.001
HbAlc (%, M_50_[P_25_,P75])	7.74 (5.77,9.62)^a^	8.30 (6.21,12.75)^a^	5.64 (4.91,6.42)	43.596	<0.001
Fins (mU/L, M_50_[P_25_,P75])	8.14 (6.63,10.06)^a^	8.23 (6.87,9.76)^a^	5.78 (5.31,6.28)	65.174	<0.001
IL-6 (pg/mL, M_50_[P_25_,P75])	10.03 (5.05,17.25)^a^	12.65 (6.07,21.80)^a^	4.53 (2.40,11.90)	16.880	<0.001
TNF-α (pg/mL, M_50_[P_25_,P75])	2.06 (1.01,3.41)	3.65 (1.23,5.14)^ab^	1.36 (0.97,3.14)	14.130	0.001
CRP (mg/L, M_50_[P_25_,P75])	0.50 (0.50,0.98)^a^	1.40 (0.50,4.55)^ab^	0.60 (0.50,1.20)	19.696	<0.001

*BMI, body mass index; LDL-C, low-density lipoprotein cholesterol; HDL-C, high-density lipoprotein cholesterol; PBG, fasting blood glucose; 2-h PG, 2-h postprandial blood glucose; HbAlc, glycosylated hemoglobin; Fins, fasting insulin; IL-6, interleukin-6; TNF-α, tumor necrosis factor-α; CRP, C-reactive protein. ^a^P, compared with the control group, P < 0.05. ^b^P: compared with the T2DM group, P < 0.05*.

### Comparison of Serum β2-GPI/oxLDL Values in Three Groups

The level of serum β2-GPI/oxLDL in the control group, in the T2DM group, and in the DCI group was 0.79 (0.57, 1.03), 1.09 (0.88, 1.28), and 1.34 (1.03, 1.68) mmol/L, respectively. The difference was statistically significant among the three groups (*Z* = 53.504, *P* < 0.001). Compared with that in the control group, the concentration of serum β2-GPI/oxLDL in T2DM and DCI groups increased significantly (*P* < 0.001). Compared with that in the T2DM group, the level of serum β2-GPI/oxLDL in the DCI group was significantly increased (*P* < 0.05), as shown in Table 2.

**Table 2:** Comparison of clinical data in three groups $\bar{x}\pm s$.

Indexes	T2DM group (*n* = 60)	DCI group (*n* = 56)	Control group (*n* = 60)	Z	*P* value
β2-GPI/oxLDL	0.79 (0.57, 1.03)^a^	1.09 (0.88, 1.28)^ab^	1.34 (1.03, 1.68)	53.504	<0.001

*^a^P, compared with the control group, P < 0.05. ^b^P, compared with the T2DM group, P < 0.05*.

### Correlation of Serum β2-GPI/oxLDL With Blood Lipids, Blood Glucose Levels, and Inflammatory Markers

The result of Pearson’s correlation analysis showed that serum β2-GPI/oxLDL was positively correlated with total cholesterol, triglyceride, PBG, 2hPG, HbAlc, IL-6, and TNF-α (*P* < 0.05), as shown in Table 3.

**Table 3:** Correlation of serum β2-GPI/oxLDL concentration with blood lipids, blood sugar, and inflammatory markers.

	Total cholesterol	Triglycerides	LDL-C	HDL-C	PBG	2 h PG	HbAlc	Fins	IL-6	TNF-α	CRP
*r*	0.176	0.288	0.043	−0.098	0.331	0.273	0.195	0.111	0.207	0.159	0.084
*P* value	0.020	<0.001	0.570	0.197	<0.001	<0.001	0.010	0.142	0.006	0.035	0.266

### Logistic Model Analysis of Clinical Factors Affecting Cerebral Infarction

Taking cerebral infarction as a dependent variable (occurrence = 1, no occurrence = 0), the basic clinical data were included in the univariate logistic model analysis, and the results showed that HbAlc, serum TNF-α, and β2-GPI/oxLDL were closely related to the occurrence of DCI (*P *< 0.05). The above independent variables were included in the multivariate model analysis, and the results showed that elevated serum TNF-α and β2-GPI/oxLDL levels were independent risk factors for DCI (*P *< 0.05), as shown in Table 4.

**Table 4:** Univariate and multivariate logistic model analyses of clinical factors affecting cerebral infarction.

Variable	Univariate		Multivariate	
	*OR* (95%*CI*)	*P*值	*OR* (95%*CI*)	*P* value
Age	0.998 (0.975–1.022)	0.866	–	–
Male	0.957 (0.456–2.008)	0.906	–	–
Smoking history	0.947 (0.436–2.059)	0.891	–	–
History of alcohol intake	0.978 (0.392–2.438)	0.962	–	–
BMI	1.036 (0.834–1.288)	0.748	–	–
T2DM course	0.562 (0.257–1.231)	0.150	–	–
Coronary heart disease	0.976 (0.466–2.044)	0.948	–	–
Arrhythmia	1.446 (0.691–3.023)	0.327	–	–
Hypertension	1.044 (0.390–2.796)	0.931	–	–
Hyperlipidemia	0.884 (0.359–2.176)	0.788	–	–
Total cholesterol	0.962 (0.700–1.321)	0.810	–	–
Triglycerides	1.525 (0.611–3.804)	0.366	–	–
LDL-C	0.890 (0.591–1.339)	0.575	–	–
HDL-C	0.282 (0.053–1.508)	0.139	–	–
PBG	1.013 (0.843–1.217)	0.892	–	–
2-h PG	0.997 (0.930–1.068)	0.929	–	–
HbAlc	1.098 (1.003–1.202)	0.042	1.076 (0.974–1.189)	0.151
Fins	0.980 (0.872–1.102)	0.742	–	–
IL-6	1.025 (0.993–1.058)	0.131	–	–
TNF-α	1.303 (1.090–1.559)	0.004	1.278 (1.047–1.560)	0.016
CRP	1.031 (0.992–1.0741)	0.126	–	–
β2-GPI/oxLDL	4.662 (1.752–12.406)	0.002	5.277 (1.815–15.344)	0.002

*BMI, body mass index; LDL-C, low-density lipoprotein cholesterol; HDL-C, high-density lipoprotein cholesterol; PBG, fasting blood glucose; 2-h PG, 2-h postprandial blood glucose; HbAlc: glycosylated hemoglobin; Fins: fasting insulin; IL-6: interleukin-6; TNF-α: tumor necrosis factor-α; CRP: C-reactive protein;β2-GPI/oxLDL: β2-glycoprotein I/oxidized low-density lipoprotein*.

### Diagnostic Efficacy of Serum β2-GPI/oxLDL for DCI

**Figure 1:**
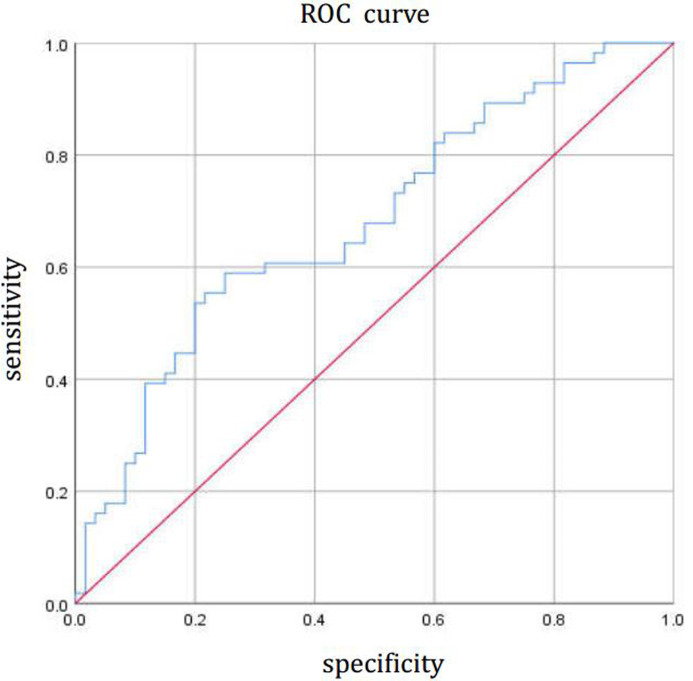
ROC curve of serum β2-GPI/oxLDL in the diagnosis of DCI.

According to the ROC curve analysis, the area under the curve of serum β2-GPI/oxLDL and their combination in the diagnosis of DCI was 0.680 (0.583–0.777), the sensitivity was 0.589, the specificity was 0.750, the cutoff value was 1.256, and the Youden index was 0.339 (*P *< 0.05), as shown in Figure 1.

## Discussion

Studies have reported that diabetes is an independent risk factor for cerebral infarction (9). The results of this study showed that serum β2-GPI/oxLDL was closely related to the occurrence of cerebral infarction in patients with T2DM. An elevated serum β2-GPI/oxLDL level is an independent risk factor for DCI, and serum β2-GPI/oxLDL is also related to lipids and inflammatory factors in DCI patients. The persistent inflammatory response may be the pathological basis of DCI. In addition, the detection of serum β2-GPI/oxLDL levels is helpful for the diagnosis of clinical DCI.

Cerebral infarction is a common complication of T2DM, with acute onset and high mortality. At present, the diagnosis of DCI mainly relies on examination by imaging, but patients are generally in the attack stage when imaging examinations are performed. Therefore, minimally invasive and accurate biological indicators for the diagnosis of DCI are of great value in patients with T2DM. Hyperglycemia can lead to changes in blood rheology (e.g., reduced red blood cell deformability, increased platelet viscosity, etc.) in patients with T2DM, leading to microcirculation disturbances and a higher risk of stroke (10). In addition, high glucose, high fat, and other risk factors can promote the occurrence of atherosclerosis (AS), which is a complex inflammatory disease and the pathological basis of cardiovascular and cerebrovascular diseases. Hyperglycemia can lead to the production of reactive oxygen species (ROS). Glucose reacts with blood proteins to form glycation end products. It can also trigger the production of ROS. ROS can trigger a chain reaction leading to increased inflammation, chemical modification of lipoproteins, and reduced nitric oxide utilization, thereby increasing the risk of atherosclerosis in the blood vessels and in the brain (11).

AS is a disease characterized by foam cell formation, lipid accumulation, and inflammation (12). Several studies have demonstrated that oxLDL, endothelial dysfunction, and oxidative stress are the most prominent risk factors for AS (13). oxLDL plays a central role in the initiation and progression of atherosclerosis, as it mediates the promotion of several vascular cells (e.g., neutrophils, monocytes/macrophages, smooth muscle cells, endothelial cells, and platelets). Inflammatory and pro-regulatory effects (14). The primary mechanism of macrophage formation stems from disturbances in oxLDL uptake and lipid efflux. Under normal circumstances, plasma low-density lipoprotein exists in the blood. Under pathological conditions, LDL-C in the plasma passes through the damaged endothelium and enters the subintima of the blood vessel, where it is oxidized by reactive oxygen species to form oxLDL. oxLDL is toxic to cells and induces inflammatory gene expression that promotes foam cell formation. Pretreatment with ox-LDL can induce downregulation of human cord blood endothelial cell viability or activate cells to secrete chemical factors, cytokines, and inflammatory factors that promote early atherosclerotic plaque formation (15, 16). The rupture of unstable plaques is the direct cause of cerebral infarction, and elevated serum ox-LDL is a risk factor for the formation of carotid atherosclerotic plaques in patients with AS cerebral infarction (17).

β2-GPI is a highly glycosylated plasma protein. β2-GPI can bind to lipoproteins and participate in lipid metabolism. In patients with autoimmune diseases (such as systemic lupus erythematosus), β2-GPI can activate systemic lupus erythematosus—Th17 and Th1 responses in atherosclerotic lesions in patients with antiphospholipid syndrome—and affect the release of inflammatory factors such as IL-17, IL-12, etc., thereby affecting disease progression (18). The increase of anti-β2-GPI antibodies can accelerate the formation of AS plaques in ApoE-/- mice, and β2-GPI can bind to negatively charged oxLDL to form a complex. In patients with antiphospholipid syndrome, the oxLDL/β2-GPI complex was found to be a predictor of heart disease. The occurrence of vascular complications has a favorable effect and is a more substantial indicator (19). The oxLDL/β2-GPI/anti-β2-GPI antibody complex increases the conversion of macrophages to foam cells, so the oxLDL/β2-GPI/anti-β2-GPI antibody complex increases gradually with the progression of AS disease. Xie et al. (20) also demonstrated that elevated β2-GPI/oxLDL and oxLDL levels were independently associated with diabetic microvascular complications. The results of our study showed that serum TNF-α and IL-6 were significantly increased in DCI, and the serum TNF-α level was an independent risk factor for DCI, which indicated that persistent inflammatory response was closely related to the occurrence of DCI. In addition, serum β2-GPI/oxLDL was significantly increased in DCI patients, and serum β2-GPI/oxLDL was positively correlated with total cholesterol, triglyceride, PBG, 2hPG, HbAlc, IL-6, and TNF-α, indicating that serum β2-GPI/oxLDL can reflect the lipid status and inflammatory status of DCI patients to a certain extent.

In conclusion, serum oxLDL/β2-GPI levels were significantly increased in DCI patients, and serum oxLDL/β2-GPI was an independent risk factor for the occurrence of DCI. In addition, serum β2-GPI/oxLDL levels can also reflect the lipid metabolism status and inflammatory status of DCI patients to a certain extent. Persistent inflammatory response and lipid disturbance may be the pathological basis of DCI. Serum oxLDL/β2-GPI has the potential to become a biomarker for the diagnosis of DCI in T2DM patients and is worthy of clinical promotion.

## Data Availability

The original contributions presented in the study are included in the article/Supplementary Material; further inquiries can be directed to the corresponding author/s.
